# Ulceration and Perforation of the Stomach

**Published:** 1852-04

**Authors:** C. S. Mann

**Affiliations:** Stoughton, Massachusetts


*Ulceration and Perforation of the Stomach.* By C. S. Mann, M. D., of Stoughton, Massachusetts.—Miss D. W., of Braintree, was a healthy and particularly strong girl at the age of 14 years, when, on the occasion of a hog being killed at her father's house, she undertook to hold up one end of a stick upon which half the hog was suspended. While doing this, she experienced a sensation "as though her stomach-bone was broken, and something gave way," as she described it. She did not suffer much inconvenience, however, until two days afterwards, when, after walking five miles and getting her feet wet, she was attacked with chills, and severe pain in the region of the stomach. From that time her health and strength decayed to such an extent that she was unable to do much work, and suffered much pain for six years. During the seventh year her health improved, and for a year, or perhaps eighteen months, she was able to do a considerable amount of work. At the expiration of that time her pain and a long train of other symptoms returned with greater severity than before. Now and then she would improve for a few months, but the relapses into suffering were frequent. In April, 1849, sixteen years after the affair of the pig, I was called to her. She had been under the treatment of some eight different physicians at various times. I found her thin, weak, suffering paroxysms of severe pain in the abdomen, at irregular intervals and of irregular duration, averaging, perhaps, once in three days, and continuing from twelve to twenty-four hours. The pulse about 120; tongue coated; breath offensive; appetite for food very small; bowels extremely costive, so that dejections occurred only at periods of eight or nine days, and there would often be discharges of considerable quantities of mucus when the constipation had been overcome by powerful cathartics. During the paroxysms of pain she had a sensation of beating in the region of the umbilicus. Moderate pressure upon a spot an inch to the left of the umbilicus brought to the fingers a strong pulsation, which was synchronous with that of the artery at the wrist. This pulsation I could always feel, whenever I examined, as long as she lived, although she did not perceive it herself except when in pain. In addition to this, there was complaint of a bearing-down sensation in the abdomen, "as though something hung down from here"—she would say, putting her hand on the region of the stomach; and she stooped in walking across the room, and frequently held her hand tightly upon the upper part of the abdomen, saying that she believed she "should fall in pieces." There was also occasional nausea.

Being unable to form a satisfactory diagnosis, supposing that there existed some important organic lesion, but having no definite idea what it might be, I directed my attention to the mitigation of some of her obvious troubles. I soon found that the inhalation of ether, practiced with the utmost caution, would relieve the paroxysms of pain, and her delight at this was worth witnessing; the use of laxatives, injections, cracked wheat, &c., overcame the constipation in some measure; carbonate of iron and quinine improved her strength, so that at the end of six months she was much better than for several years before. She had a year and a half of tolerable comfort. She did considerable work about the house, and had the paroxysms of pain unfrequently, and only after some error in diet. The medicine which seemed to produce more direct benefit than any other, and which was given at a period some nine months, I think, after I began to attend her, was acetate of lead, administered in very small doses and continued for two or three weeks. The pulse had varied from 105 to 120 until this time, when it came down to 90, and remained during nearly a year from 90 to 100. On Thursday, April 24th, 1851, I was called to her, and found her with the same symptoms which she had frequently exhibited, viz: pain, nausea and weakness. I found that she had neglected the use of injections, &c., and that the constipation had returned. The inhalation of a few drops of ether overcame the violence of the pain, and I left pills of aloes and myrrh, and ordered repeated injections. I saw her again on the 26th, in the afternoon, and found her more ill. Only one slight dejection had resulted from the pills, and nausea and occasional vomiting, with pain, had kept her sleepless during the previous night. Not liking to give her an emetic, I gave fluid extract of valerian and sup. carbonate of soda; but the taste of the valerian excited vomiting, and she rejected perhaps a pint and a half of dark-colored fluid mixed with mucus. This seemed to relieve her. She became cheerful and talkative, and I left her with the promise of calling on the next morning, and expected to find her comfortable. At about 8, the next morning, a messenger came in haste, saying that she was very sick. She had passed a comfortable night, and seemed convalescent at 7, in the morning. At about that time, however, while reaching her hands forward to pull towards her a comforter upon her bed, which was not arranged as she wished, she had a sensation of "something giving way and falling down," and of excruciating pain. I found her looking badly, pulse very quick, small and weak, thirst great, with much anxiety of mind. The abdomen hard. Opium and powerful stimulants produced but little effect, and she died at 9, in the morning.

An autopsy was made, seventeen hours after death, in the presence of Drs. Fifield, father and son, of Weymouth; Dr. Bugbee, of Quincy, and Dr. Holmes, of Braintree. The cavity of the abdomen contained perhaps a quart of fluid, a part of which was pus. Upon the anterior part of the stomach was an opening as large as a dime, with only the slightest remnant of a thin membrane which had covered it. The walls of this ulcer were of the thickness of two thirds of an inch, and its breadth perhaps two inches. A patch of highly-inflamed mucous membrane, of the size of a quarter of a dollar, bordered upon this thickened portion. The peritoneum was considerably injected. The bladder was contracted to the capacity of not more than a gill. There had been occasional complaints of difficulty in passing the urine, and of the quantity of it being too small, but not such as to lead me to think this to constitute an important feature in the case.

I suppose that the effort of lifting the hog ruptured one of the coats of the stomach, and that the attempts of nature to heal the part have kept up an inflammation ever since, about eighteen years. All the symptoms, excepting the pulsation, are satisfactorily accounted for, and as there was no abnormal condition of the heart or arteries apparent in a very hasty examination of them, this must pass for an increased action of the abdominal aorta.—*Boston Med. & Surg. Journal*, 1852.

